# Hernia

**Published:** 1894-09

**Authors:** 


*Hernia.* By THOMAS H. MANLEY, M.D. Pp. 231. London: F. J. REBMAN. 1893.

We have derived considerable amusement from a perusal of this book, which in many respects is truly original.

The author informs us at the outset that "there are but few American treatises on the subject of hernia. . . . The views, opinions, and deductions in this book are based on a moderate experience in private and hospital practice, and on what could be gathered through a diligent perusal of the latest literature, domestic and foreign."

We can best give an idea of the book by a few extracts:—

"There are but few things necessary in operation for strangulation. They are: 1st. Good light. (At night I prefer candlelight to any other.) 2d. Plenty of pure water. 3d. A warm, clean room. 4th. Assistants, medical, if possible; one at least, if not one or two, intelligent, *cool-headed* women." The italics are the author's. "The technique of operations for strangulation *have*, during the past fifty years, been enormously simplified." The italics are ours. (Both on page 190.) "But, in the inguinal hernia with a woman, as all her generative system is internal, a prolapse of any or many appendages of it, often *make their way* into an inguinal hernial sac." (p. 206.) "Pathological hernia are common." (p. 210.) "Solicitious." (p. 18.) "Psycical." (p. 208.)

A tragic episode is recorded of a house-surgeon, operating for strangulated hernia, who "removed a wash-basin full of intestine, and then found it impossible to dam back the fæces or bring the separated edges together."

We can promise the reader of this book that he will not find a dull page from beginning to end.

